# Attitude Toward Mathematics of Future Teachers: How Important Are Creativity and Cognitive Flexibility?

**DOI:** 10.3389/fpsyg.2021.713941

**Published:** 2021-07-09

**Authors:** Cristina de-la-Peña, Raquel Fernádez-Cézar, Natalia Solano-Pinto

**Affiliations:** ^1^Departamento de Psicología Evolutiva y Psicobiología, Universidad Internacional de la Rioja, Logroño, Spain; ^2^Department Mathematics, Universidad de Castilla-La Mancha, Ciudad Real, Spain; ^3^Psychology Department, Universidad de Castilla-LaMancha, Ciudad Real, Spain

**Keywords:** attitude toward mathematics, creativity, cognitive flexibility, higher education, cognitive processes

## Abstract

The attitude toward mathematics is shaped by cognitive components such as beliefs and cognitive processes. However, the importance of cognitive processes in attitude toward mathematics has not yet been researched. Therefore, this study aimed to identify the role of cognitive processes, creativity and cognitive flexibility, in the attitude toward mathematics of future teachers. For that purpose, 218 University students and preservice teachers, completed assignments on creativity and cognitive flexibility and a questionnaire on attitude toward mathematics. The results showed that the use of innovative details (a creativity subscale) rises the probability of exhibiting a positive attitude toward mathematics by 1.81. Besides, cognitive flexibility rises this probability by 2.32. The conclusion is that both, details and cognitive flexibility act as good predictors of a positive attitude toward mathematics. This has implications for educational practice in the planning of mathematics instruction in higher education, specifically, in future teachers.

## Introduction

The attitude toward mathematics is a topic that generates interest on the part of researchers due to the influence it has on student learning and performance in this subject (Pekrun et al., 2017; Sanchal and Sharma, 2017). In the scientific literature (Han and Carpenter, 2014), the attitude toward mathematics is formed by cognitive, affective and behavioral components that interact with each other. As for the cognitive component, reference is made on the one hand to beliefs about mathematics and, on the other hand, to the cognitive processes involved in the attitude toward mathematics. This research focuses on two cognitive processes that other studies (Fetterly, 2020; Riling, 2020) associate with the mathematical context, such as creativity and cognitive flexibility. Therefore, this study aimed to identify the role of creativity and cognitive flexibility in the attitude toward the mathematics of University students and future teachers.

## Cognitive Processes in the Attitude Toward Mathematics

In the scientific literature, there are different definitions of attitude. Han and Carpenter (2014) establish that attitude is an affective, cognitive, and behavioral reaction to an environment or object, while for Rodríguez et al. (2020) attitude toward mathematics refers to beliefs about the effectiveness and interest of students in performing mathematical tasks in academic and everyday situations. In this study, the attitude toward mathematics is the disposition of a person toward a mathematical task resulting from his/her way of thinking, feeling, or acting.

Regarding the development of attitude, Han and Carpenter (2014) indicate that attitude toward mathematics is formed by cognitive, affective, and behavioral components that interact with each other. According to these authors, the cognitive component comprises what is believed or thought about mathematics, the affective component is the feeling or emotion toward mathematics, and the behavioral component is the tendency to respond to mathematical learning. In this line, the models of attitude toward mathematics (Abraham et al., 2010; Di Martino and Zan, 2015) obtained through qualitative methods confirm the participation of both cognitive and non-cognitive (affective and behavioral) components. The cognitive component of the attitude toward mathematics is consisting of, on the one hand, the beliefs about mathematics, and on the other hand, the cognitive processes involved in that. Beliefs about mathematics have been widely studied in the literature. They refer to psychologically-based propositions about mathematics that are believed to be true (Philipp, 2007). Nonetheless, the cognitive processes involved in the attitude toward mathematics have been scarcely studied. The research found relates the attitude toward mathematics to reasoning skills (Lipnevich et al., 2016), decision-making (Rolison et al., 2020), cognitive reflection (Morsanyi et al., 2014), and creativity (Sharma, 2014). Specifically, in the mathematical context, researchers pay more attention to creativity, which is considered as an action in the Creative Mathematics Action Framework (Riling, 2020) and as a thought process or product manifested in fluidity, cognitive flexibility, and originality (Fetterly, 2020). Therefore, creativity is connected to the attitude toward mathematics through actions involving mathematics. However, up to date, there is a lack of models explaining how creativity, as a cognitive process that is part of the cognitive component of the mathematical attitude, influences this attitude toward mathematics. Besides, research is found such as Mann (2005) study, which finds an association between positive attitude toward mathematics and greater creativity, and Nijstad's two-way model of creativity, and Nijstad et al. (2010) reflect that the activation of positive moods enhances creativity through the stimulation of cognitive flexibility. In this model (Nijstad et al., 2010), one of the ways to be creative is through cognitive flexibility, emphasizing that a key component of creativity is cognitive flexibility or the ability to adopt different perspectives, an approach also supported by other authors (Vartanian, 2009; Zabelina and Robinson, 2010; Haavold, 2021).

In the mathematical context, cognitive flexibility is a critical issue in problem solving. Clément (2006) understands it as the ability to select from various perspectives or strategies. Heinze et al. (2009) reflect the relevance of cognitive flexibility in interpreting situations or in using mathematical strategies, and Vartanian (2009) evidence that cognitive flexibility confers advantages on creative people in problem solving because changes in the structure of a problem require the necessary adjustments in problem-solving strategies. The theory of cognitive flexibility (Spiro et al., 2003) emphasizes that cognitive flexibility is necessary to address a mathematical concept (Jonassen, 2011), and as a teacher, cognitive flexibility facilitates the creation of learning scenarios considering different perspectives (Valentine and Kopcha, 2016).

In this study, we focus on University students and specifically on future teachers. The reason is that these students will be teaching mathematics in their future lives, and the mathematics instruction they will carry out will be influenced by their attitude toward mathematics (Raymond, 1997) and will subsequently affect the perception of mathematics of their future students and so their achievement (Bekdemir, 2010; Cardetti and Truxaw, 2014) and performance in the subject. Some studies show the connection between mathematics teaching and the attitudes of students toward this subject (Rojas and Deulofeu, 2015) and the influence of the attitude of the mathematics teacher on the performance of the student in mathematics (Desimone, 2009). Regarding the specific results of the research carried out with University students, Bates et al. (2011) find that the future teachers are the University group with more negative attitudes toward mathematics and Cargnelutti et al. (2017) show the presence of mathematical anxiety in the future teachers, that this persists over time, and negatively influences student learning and performance.

Research has shown that basing mathematics education on transmission leads to limited and biased conceptions in this field. This has generated an interest in creating learning environments where mathematical concepts and processes are transmitted (Kisa and Stein, 2015). Dimmel and Herbst (2017) point out the need for a positive attitude toward mathematics among teachers to incorporate active and innovative learning scenarios in their classrooms. In this line, we consider it crucial for future teachers, on the one hand, to be aware that his or her attitude toward mathematics affects the attitudes and/or performance of students, and on the other, that the mathematical attitude is made up of cognitive components that can be known and promoted from their parts, such as creativity and cognitive flexibility. With this knowledge, future teachers would design instructional proposals by using their creativity and cognitive flexibility, which will be aimed at students having positive experiences with mathematics and so improving the mathematical attitude and performance of students.

This research hypothesizes that the cognitive components (creativity and cognitive flexibility) are related to the attitude toward mathematics in a specific population such as University students who become mathematics teachers. In this study, tests were used to evaluate creative potential and cognitive flexibility from a generic point of view, without directly referring to mathematical competencies. The aim was to use simple tests with stable psychometric properties for the general population, without being conditioned to a certain level of mathematical competence. In addition, in the case of creativity, tests evaluating divergent thinking based primarily on the originality and innovation of proposals have been reviewed as a way of assessing the creative potential. This way of evaluating creativity is considered to be a predictor of future success even if one has no solid knowledge in a particular field (Runco and Acar, 2012).

## Methods

### Participants

The sample consisted of 218 University students (77.1% women and 22.9% men, with an age range of 17–47 years, *M* = 19.7, *SD* = 2.71). Out of that, 114 were first-year students and 104 were second-year students. All the participants have taken mathematics subjects in addition to other subjects in the education major. The inclusion criteria were to be students of Education, to be over 18 years old at the time of taking the test, to be enrolled in a mathematics-related subject, and not to have taken the tests previously.

### Materials

The instruments applied in the research were:

Modified Attitude toward mathematics scale (Auzmendi, 1992; Fernández-Cézar et al., 2016). It is a test composed of 10 items that evaluate the degree of agreement and disagreement by a 5-point Likert scale. The items are statements dealing with thoughts and emotions toward mathematics. The internal consistency evaluated with Cronbach's α offered by the authors is 0.91 (Fernández-Cézar et al., 2016), which coincides with the obtained sample of this study.

Creative Imagination Test for Adults (PIC-A) (Artola et al., 2012) assesses the creative potential of students through four tasks and provides information on eight scales (fluency, flexibility, narrative originality, fantasy, graphic originality, elaboration, special details, and title) and three creativity indexes (narrative, graphic, and general). The meaning of scales and indexes is as follows: fluidity, as the capacity for ideas production; flexibility, as the capacity to produce answers varied in type and topics; narrative originality, as innovative narrative answers; graphic originality, as innovative answers from a graphic representation; fantasy, referred to the generation of ideas from offered data; elaboration, as the descriptive presentation of the ideas; special details, as the use of innovative details; title, as the capacity to put special titles to the narrated or represented ideas; narrative creativity (innovative verbal proposals composed of the fantasy, fluidity, flexibility, and creative originality scales), and graphic creativity (innovative non-verbal proposals composed of the graphic originality, elaboration, special details, and title scales), and the general creativity index (sum of narrative and graphic creativity, and indicator of the creative potential to generate new and original ideas). The internal consistency (Cronbach's α) offered by the authors is 0.83, while it is 0.78 for this study sample.

Changes Test (Seisdedos Cubero, 1994) assesses the cognitive flexibility in adults through a simple geometric figure task in which the response must match under the change of different parameters. The internal consistency for this sample (Cronbach's α) is 0.87.

### Procedure

About 3 days were scheduled to administer the tests that were completed in the respective classrooms of students. There were three class groups and each student was delivered a paper test. First, they completed the creativity task, which was followed by the cognitive flexibility and attitude toward mathematics tests, respectively. The time taken for the test in each classroom was approximately 70 min and the first author was in charge of administering the tests to the students. Prior to taking the test, all the University students (adults) signed written consent, were informed that the participation was voluntary and had withdrawal possibility at any stage. All the data were collected in compliance with the ethical guidelines of the Helsinki Declaration and the confidentiality of the data was guaranteed. The research was revised and approved by the Asociación Educar para el Desarrollo Humano Ethics Commission.

### Data Analysis

The Social Science Statistical Package, SPSS (Windows version 25), was used to carry out the analyses. Descriptive statistics were used to find out the means and SD of the studied variables. Provided the sample size, the Kolmogorov-Smirnoff test was used for normality, obtaining *p* < 0 for all of them which requires the use of non-parametric coefficients and inferential test (López-Roldán and Fachelli, 2015; Montgomery, 2017). Therefore, to obtain the correlations between the variables, the Spearman coefficient was used. The variables, cognitive flexibility (evaluated with Changes Test) and creativity components (evaluated with PIC-A Test) were then categorized by taking into account the mean plus half the SD as the cutoff point. Finally, a binary logistic regression was performed with attitude toward mathematics as the dependent variable, to identify its predictors among the studied variables. Hosmer-Lemeshow test was considered for model goodness-of-fit.

## Results

The first step was to carry out the descriptive analysis by means of central tendency and dispersion indexes of the variables, obtaining that the cognitive flexibility task was within the average according to the scales of the test (Seisdedos Cubero, 1994). The attitude toward mathematics at a moderately negative level and the general creativity and creativity indexes at a low level in comparison with the scales were offered by the authors of the assessment instrument (Artola et al., 2012; Table 1).

**Table 1:** Mean (M) and SD for cognitive flexibility, mathematical attitude, creativity, and matrix correlation.

	**Mean** ***(M)***	**Standard deviation** ***(SD)***	**Correlations with mathematical attitude**
Cognitive flexibility	13.20	6.30	−0.251^**^
Mathematical attitude	29.40	9.12	1
Fantasy	5.22	3.23	0.122
Fluency	30.63	10.42	−0.024
Flexibility	18.40	4.88	−0.017
Narrative originality	6.47	4.34	−0.083
Graphic originality	1.99	1.62	−0.013
Elaboration	2.03	1.67	−0.053
Details	2.11	1.72	−0.135^*^
Title	0.803	1.28	−0.070
Narrative creativity	58.59	19.45	−0.033
Graphic creativity	6.90	4.29	−0.098
Total creativity	65.46	21.04	−0.049

* *p < 0.05*;

** *p < 0.001*.

The next step was to examine the relationship between attitude toward mathematics and flexibility and creativity finding that mathematical attitude had a negative and statistically significant relationship with cognitive flexibility (*r* = −0.251, *p* < 0) and details (*r* = –0.135, *p* < 0.05; Table 1). To analyze the effect of cognitive-affective variables on the attitude toward mathematics, taking into account that the independent variables do not meet normality criteria, we conducted a binomial logistic regression analysis with attitude toward mathematics as the categorical outcome and the high level as the contrast group. Cognitive flexibility and details were included as predictors, assuming the high level as the reference. The descriptive statistics of categorized variables are presented in Table 2. The differences in attitude toward mathematics between the high-low groups are significant (*U* = 10.65, *p* < 0, *d* = 1.66). In addition, the value of the effect size is high (Cohen's *d* > 0.80), being able to state that the differences are due to high-low groups (Cohen et al., 2018).

**Table 2:** Mean (M) and SD of categorized variables by media and plus half SD.

	**Low level**		**High level**	
	**Mean** ***(M)***	**Standard deviation** ***(SD)***	**Mean** ***(M)***	**Standard deviation** ***(SD)***
Cognitive flexibility	9.51	3.70	20.43	3.39
Creativity				
Fantasy	3.83	1.88	9.90	2.28
Fluency	29.79	9.28	60.16	2.13
Flexibility	16	2.94	24.18	3.63
Narrative originality	5.88	3.8	13	4.3
Graphic originality	1.82	1.38	6.3	1.06
Elaboration	1.70	1.20	6.18	1.04
Details	0.59	1.04	6.25	1.50
Title	0.70	1.04	6.25	1.50
Narrative creativity	47.77	10.98	81.48	12.25
Graphic creativity	3.25	1.42	9.62	3.65
Total creativity	53.73	11.64	90.78	12.88

The Omnibus test is significant, which indicates that the dependent variable (attitude toward mathematics) is explained by at least one variable (χ^2^ = 12.78, *p* < 0.05). The results of the analysis are presented in Table 3, including odds ratio (ORs) and the 95% of confidence interval for each predictor. ORs reflect the increase (or decrease) in the odds of a participant being in the contrast group relative to the high attitude toward the mathematics group based on the change among categories for each predictor. In the predictors, the high category has also been taken as the reference.

**Table 3:** Model of logistic regression for the attitude toward mathematics.

	**B**	**Wald**	**Sig**	**Exp (B)**	**IC 95% Exp (B) lower-higher**
Cognitive flexibility^*^	−0.843	7.949	0.005	0.430	0.240–0.773
Details* (subescale of creativity)	−0.595	4.478	0.039	0.552	0.318–0.957

* *p < 0.05*.

The Hosmer-Lemeshow value is 0.59, well above 0.05, exhibiting more than a reasonable goodness-of-fit (χ^2^ = 1.05, *p* = 0.59). In the proposed model, the chance of participants belonging to the high attitude toward mathematics group is 61%.

We will illustrate the interpretation of the OR for the cognitive flexibility in the comparison of the high to the low attitude toward mathematics groups, from data in Table 3. The OR represents the change in the odds of being in the low compared to high attitude toward mathematics group, which mathematically is got as 1/expB. In this case, it is 2.32 (1/0.430) to cognitive flexibility which means that the odds for participants with low cognitive flexibility to belong to the low attitude toward mathematics group was 2.32 times more likely than for participants with high cognitive flexibility. In the same way, 1.81 (1/0.552) to details (subscale of PIC-A and creativity test) means that a participant with a low score in details is 1.81 times more likely to have a low attitude toward mathematics than a participant with a high score in details (use of innovative details).

## Discussion

This study aimed to identify the role of cognitive processes, creativity, and cognitive flexibility, in the attitude toward the mathematics of future teachers. For that purpose, 218 University students and preservice teachers completed assignments on creativity and cognitive flexibility and a questionnaire on attitude toward mathematics.

The results confirm the relationship between creativity (use of innovative details) with mathematical attitude in future teachers. These results are in the direction of those found by Mann (2005) for creativity which does indicate a relationship with the attitude toward mathematics.

Furthermore, in this study, a significant negative relationship has been obtained between the attitude toward mathematics and cognitive flexibility. Specifically, the study indicates that those students who have a negative attitude toward mathematics are less able to adopt different perspectives on mathematical facts, situations, or problems. The connection between cognitive flexibility and the attitude toward mathematics is a novel finding that must be deeply explored in future research.

In this sense, the data obtained are congruent with those provided by other investigations that show the relevance of cognitive flexibility in problem solving (Clément, 2006; Heinze et al., 2009; Vartanian, 2009; Cragg and Gilmore, 2014). An interpretation of that could be that cognitive flexibility allows adaptation and problem solving in an adaptive way, a relevant aspect in the work of the teacher. The results suggest that cognitive flexibility is a variable to be considered in the attitude toward the mathematics of future teachers. In this direction, authors such as Dimmel and Herbst (2017) and Valentine and Kopcha (2016) suggest that to teach mathematics actively and innovatively, future teachers need cognitive flexibility and a positive attitude toward mathematics. Therefore, by promoting the cognitive flexibility of future University teachers, a more positive attitude toward mathematics can be fostered, and, consequently, in the long run, the transmission to their pupils can be achieved through the creation of different learning scenarios that affect the attitude of students toward the subject of mathematics (Rojas and Deulofeu, 2015).

As pointed out by different authors, cognitive flexibility is the ability to select various strategies in solving problems, a critical issue in the mathematics context (Clément, 2006; Heinze et al., 2009; Vartanian, 2009). Thus, in this research, we consider that for a future teacher, the more creative and flexible they are in formulating mathematics proposals, the more positive attitude toward mathematics they exhibit. It will redound for their future students in a more effective way to get an insight into mathematics (Jonassen, 2011) which will entail a more effective performance.

The educational implication derived from this research is two-fold. On the one hand, a main theoretical implication is revealed: cognitive flexibility influences the attitude toward mathematics in future teachers. The positive attitudes of teachers, embedded in their teaching practice, can help students in the early stages of education to actively build their learning and improve their mathematics achievement. Following this line, the positive attitude of teachers toward mathematics would be reflected in, or provoke, positive emotions that contribute to the development of adaptive coping styles that, in turn, are related to the development of cognitive flexibility (Seligman et al., 2005). In this area, more research is needed. On the other hand, a significant practical implication is inferred: the planning of mathematics instruction incorporating the work on the attitudes toward the mathematics of future teachers, the consideration of cognitive flexibility in the creation of teaching activities, and the use of innovative details. All that should be intended to generate positive attitudes in a population as crucial as future teachers are because this way they could become educational models with mathematical attitudes that they will possibly transmit to their future students (Cardetti and Truxaw, 2014). The attitude is fundamental to foster mathematical competence (Dowker et al., 2019) and constitutes a learned predisposition that affects the construction of the person itself, and the knowledge, being learned and susceptibly improved. The present investigation has several limitations. Thus, due to the sampling characteristics and the lack of control of foreign variables, it is necessary to be cautious in the generalization of results. This study points toward future lines of research in which the analysis of the influence of cognitive processing on the attitude toward mathematics allows a better quality of competence in the mathematics teachers, with consequences in students in the lower educational stages. The results suggest that cognitive flexibility and the use of innovative details can predict the attitude toward mathematics. On the contrary, at least in the sample of this study and with the assessment instruments used, it seems that creativity (except for the details subscale) is not a cognitive variable that can predict such attitude. As future action lines, the selection of the sample randomly among different universities is pointed out, and the control of different variables could be predictive of the mathematical attitude. Although in this study the evaluation of creativity and cognitive flexibility was intended generically, in future studies, the use of tests associated with the mathematical field can be considered, creating in such case homogeneous subgroups in relation to the mathematical competence to contrast possible differences in the cognitive variables. For example, other cognitive variables such as the perceived self-efficacy in mathematical tasks, the evaluation of the mathematical reasoning skills, or the creative potential in logical-mathematical thinking should be considered. In addition, a longitudinal design could be used to know how the relationship between the variables evolves throughout University teacher training courses. Therefore, taking into account the different considerations, the involvement of cognitive variables and the attitude toward mathematics in future teachers can be reflected with greater empirical accuracy.

## Data Availability Statement

The raw data supporting the conclusions of this article will be made available by the authors, without undue reservation.

## Ethics Statement

The studies involving human participants were reviewed and approved by Asociación Educar para el Desarrollo Humano Ethics Commission. The patients/participants provided their written informed consent to participate in this study.

## Author Contributions

Cd-l-P, RF-C, and NS-P contributed to design of the study. Cd-l-P collected data and corrected the filled in tests. NS-P and RF-C performed the statistical analysis. Cd-l-P wrote the first draft of the manuscript. NS-P wrote manuscript Methods and Results sections. All authors contributed to manuscript revision, read, and approved the submitted version.

## Conflict of Interest

The authors declare that the research was conducted in the absence of any commercial or financial relationships that could be construed as a potential conflict of interest.
